# Crop response to El Niño-Southern Oscillation related weather variation to help farmers manage their crops

**DOI:** 10.1038/s41598-021-87520-4

**Published:** 2021-04-15

**Authors:** Ross Chapman, James Cock, Marianne Samson, Noel Janetski, Kate Janetski, Dadang Gusyana, Sudarshan Dutta, Thomas Oberthür

**Affiliations:** 1 Data Analysis Consultant, 9 Kia Ora Parade, Ferntree Gully, VIC 3156 Australia; 2 Emeritus, Centro Internacional de Agricultura Tropical (CIAT), Cali, Colombia; 3 Scientific Data Management Consultant, Los Banos, Philippines; 4 Senior Technical Advisor to the Cocoa Care Program of Community Solutions International, Makassar, Sulawesi Indonesia; 5 CEO of Community Solutions International, Bali, Indonesia; 6 Lautan Luas TBK, Jakarta, Indonesia; 7 African Plant Nutrition Institute, Lot 660, Hay Moulay Rachid, Ben Guerir, Morocco; 8 Business and Partnership Development, African Plant Nutrition Institute, Lot 660, Hay Moulay Rachid, Ben Guerir, Morocco

**Keywords:** Computational models, Data mining, Data processing, Machine learning, Probabilistic data networks, Computational biology and bioinformatics, Climate sciences, Ecology, Agroecology, Plant sciences, Plant ecology

## Abstract

Although weather is a major driver of crop yield, many farmers don’t know in advance how the weather will vary nor how their crops will respond. We hypothesized that where El Niño-Southern Oscillation (ENSO) drives weather patterns, and data on crop response to distinct management practices exists, it should be possible to map ENSO Oceanic Index (ENSO OI) patterns to crop management responses without precise weather data. Time series data on cacao farm yields in Sulawesi, Indonesia, with and without fertilizer, were used to provide proof-of-concept. A machine learning approach associated 75% of cacao yield variation with the ENSO patterns up to 8 and 24 months before harvest and predicted when fertilizer applications would be worthwhile. Thus, it’s possible to relate average cacao crop performance and management response directly to ENSO patterns without weather data provided: (1) site specific data exist on crop performance over time with distinct management practices; and (2) the weather patterns are driven by ENSO OI. We believe that the principles established here can readily be applied to other crops, particularly when there’s little data available on crop responses to management and weather. However, specific models will be required for each crop and every recommendation domain.

## Introduction

Globally there is a need to produce more food in a sustainable manner^1,2^. Sixty percent of the annual variation in yield of several major crops is related to year to year variation in weather. The annual variation is particularly severe in rainfed agriculture, which comprises 80% of the total land area and 58% of the overall production^3^. The annual yield variation, which is closely related to rainfall patterns, is enormous; however, no overall estimates on losses because of drought and short dry spells are available^4^. Farmers are aware of the role of weather in yield variation. Farmers often monitor and predict weather and seasonal climate events through locally observed variables and make management decisions based on these forecasts and their historic knowledge of how the weather affects their crops^5^. Thus, in Indonesia, when rains are delayed, farmers take this as a sign that there is likely to be a future shortage of rainfall and reduce their rice area, whilst increasing the area planted to maize in Java and Bali^6^ or leaving a forced fallow in Sulawesi^7^. However, farmers, particularly those in the developing world, have few analytical tools they can use to better manage their crops in the face of uncertain weather patterns. Even if they have some idea of the future weather patterns for the coming months due to improved weather forecasts, they generally have inadequate information on the response of their crop to changes in the weather patterns, and hence how to optimize management of their crops according to the expected weather conditions. We explore the potential of modern information systems and analytical methodologies to help farmers manage their crops under variable weather conditions.

The weather patterns across many regions of the world are influenced by the El Niño-Southern Oscillation (ENSO), which apart from the regular progression of the seasons is the most predictable climate fluctuation on the planet^8,9^. The effect of the changes in the ENSO state on the weather varies from place to place^9^. The ENSO cycle can now be reasonably well predicted 6 months or more in advance (see for example Ham et al. 2019^10^). Thus, if the relationship between the weather and the state of the ENSO cycle at a specific location is known it should be possible to predict the weather for that site reasonably accurately.

Jones et al. (2003)^11^ indicated that better climate predictions based on the expected ENSO phase 3 to 6 months ahead of time, coupled with crop simulation models, could be used to determine the optimum crop mix in Argentina, and also to improve maize crop management (planting date, hybrid use, nitrogen fertilizer amount, and plant density). Podestá et al. (2002)^12^ followed this approach, first looking at the historical relationship between ENSO and the weather, and then using a combination of historical field data and crop simulations to explore the response of the crop to distinct management of maize, or allocation of land to other crops, under various ENSO scenarios. They concluded that more scholarly research is required on all the components of the system to implement this approach. We note that maize is one of the most highly researched crops in the world with well-developed crop simulation models^11^.

Another approach has been to use probabilistic models, rather than simulation models, to associate yield with weather and other agronomic variables. This methodology, based on statistical analysis of historical data, was used to associate yield of wheat and barley in Canada with weather and other variables^13^. The model development was totally dependent on the availability of detailed data on both crop yields and weather. Separate models were required for each of the crops. Later, an ENSO component was added to the initial model, non-linearity was considered, and spatial clusters were added to the analysis. The addition of clusters, presumably with similar environments, improved the predictability of the models^14^. The clusters are similar in principle to the earlier concept of recommendation domain, which is defined as "a group of roughly homogeneous farmers with similar circumstances for whom we can make more or less the same recommendation"^15^ or homologous zones with similar weather conditions^16^ and more recently the ‘cohorts’ of Technology Extrapolation Domains (TEDS)^17^ and an Extrapolation Domain Analysis that includes socio-economic conditions^18^. The incorporation of the ENSO component improved the predictive ability of the models possibly suggesting that certain components of the weather not captured from the data sources used in the original model were inferred from the ENSO phases. Furthermore, no two crop districts used the exact same combination of variables to predict crop yield. The model did not include crop management factors; however, the authors recognized that if this information were available the model’s predictive capacity would be improved. This probabilistic approach depends on data sets on yield under distinct weather conditions, is location or cluster specific, and mixes real data on weather variables with the indirect effects of the ENSO through its influence on the weather. If management is to be incorporated into these models, information on the response to management under distinct environmental conditions is required.

In India a whole series of models were developed to assist farmers in managing their crops. These models ranged from probabilistic models to simulations of processes such as water balance, however, all of these models were based on weather data and assistance to farmers depended on weather forecasts^19^.

In 2017 we were analysing data on the effects of a management variable, fertilizer, on the yield of cacao from a series of on farm trials in Sulawesi. There were very large annual differences in yield and the fertilizer response. These distinct annual responses were attributed to weather variation, which in turn appeared to be associated with the ENSO phenomenon^20^. Due to the lack of reliable weather data, the study concluded that when low prices and adverse weather are expected many farmers would be better off not applying fertilizer^20^. However, the study did not specify precisely which weather conditions would be considered adverse^20^.

We hypothesized that where ENSO is the driver of weather patterns and data on crop response to distinct management practices were available, it would be possible to look for direct associations between ENSO patterns, yield and response to management without precise weather data. If this hypothesis were to be correct it could provide growers of crops that have not been intensively researched in areas with poor access to accurate weather data with guidelines on how to better manage them. This would be especially true if longer term predictions of ENSO status were available.

We decided to test this hypothesis using *Theobroma* *cacao,* commonly known as cacao or cocoa, which has been shown to be sensitive to ENSO OI fluctuations^21^ and for which a reasonable data set was available on yield with variation in at least one management variable. There is no mechanistic hypothesis to determine how ENSO drives the weather in the study area nor how the weather in turn influences cacao crop development and yield. Thus, we adopted a blackbox, machine learning approach to provide proof of concept. This approach has the advantage of not requiring assumptions about the relationships but has the disadvantage of not providing information on the nature of the associations detected.

## Materials and method

### Data sources

#### ENSO

The ENSO data was the NOAA Optimum Interpolation (OI) Sea Surface Temperature which can be obtained from NOAA^22^.

#### Cacao yield data

The cacao yield data were obtained from a series of on farm yield trials set up by the International Plant Nutrition Institute in collaboration with the Cocoa Care (http://www.ecocsi.org/cocoa-care/about-cocoa-care/) program in the Soppeng district (4° 20′ S, 120° 15′ E), South Sulawesi Province, Republic of Indonesia. The data with 73 farms described in Hoffman et al. (2020)^20^ was used. While Hoffman et al. (2020)^20^ had reclassified these farms into four new groups, we used the original 10 farmer groups. Members of each group were from similar geographic regions and shared the same start and end dates for experimental observations. The farmers in the groups all attended the Mars Cocoa Academy at Tarenge, Sulawesi, to learn Good Agricultural Practices (GAP) and how to better manage their farms. GAP were applied on all farms, and each farm had at least 800 trees ha^−1^. Two adjacent blocks of 50 trees were selected from within an area that was representative of the whole farm^20^; one plot received applications of inorganic fertilizer while the other remained unfertilized. The trials for each group were monitored for yield over a period of 2 years with monthly yields computed from measurements taken every 2 weeks. The trials of the distinct groups were started at different times so not all groups could be compared over the same time period. The yield data was taken over 5 years from July 2013 until October 2018.

### Establising associations between monthly yield and ENSO

Establishing the relationships between complex patterns of the ENSO phenomenon with traditional statistical methods would require constructing and testing a statistical model based on *ex-ante* assumptions regarding the likely relationships within the data^23^. We had no basis for making such suppositions. Furthermore, statistical models that attempt to describe a complex system with many parameters, interactions and non-linear responses typically require ‘long data’ where the number of subjects is very much higher than the number of parameters^23^. On the other hand, machine learning methods require no previous knowledge or guesses at the relationships and can explore complex relationships and patterns between parameters more effectively than conventional statistical analyses, especially from ‘short data’, where the number of subjects are relatively low^23,24^.

Bayesian Neural Networks (BNN) were chosen as a machine learning method that was a priori likely to establish the desired associations. Bayesian based methods are attractive as they create a probabilistic framed output; whence, the yield probability distributions could predict the most likely level of crop yields under distinct scenarios, which is desirable when deciding on future actions. For example, the output might predict a very high probability that fertilizer applications would lead to significant increases in yield or, alternatively, that little or no yield response would arise. Such data would help reduce the uncertainty linked to crop yield responses and substantially increase the margin of confidence associated with management decisions.

The BNNs were constructed using the *Edward*^25,26^ Python library. The precision of the BNNs were tested using cross-fold validation^27^ where 10% of the dataset is randomly withdrawn and set aside as a hold-out dataset. A preliminary network is then built using the 90% of retained data. The accuracy of this preliminary network is assessed by predicting the cacao yield of the 10% hold-out data set based on other parameters and comparing the prediction with observed data. The testing process is repeated 10 times, each time using an independent and randomly selected slice of data, and the mean precision of all ten networks are used to describe the expected precision of the learning process. The accuracy with which the model predicted the values in the test data were assessed using a combination of r^2^ derived from ordinary least squares regression, and mean square error, which measures mean squared difference between the predicted and actual values. A final network is then constructed using the complete data set once architecture and accuracy validations were complete. The BNN was trained using ENSO OI data extracted from a period of 1–9 (short term) or 1–25 months (long term) prior to harvest.

Once the construction and evaluation of the BNNs were completed, the models were deployed to predict the impact of fertilizer management under three contrasting ENSO profiles that were selected to give a diverse range of weather conditions during the period leading to the cacao crop harvest. Full details of the ENSO OI values used in each ENSO profile are available in Supplementary Table S.1.

1. Continuous neutral spanning the 9 months prior to harvest (Neutral).
2. An ENSO profile spanning 9 months prior to harvest centered on the maximum observed ENSO index (2.6) (MaxCent) and incorporating the observed ENSO OI indices 2 and 4 months prior to and post the observed maximum.
3. An ENSO profile spanning 9 months prior to harvest centered on the minimum observed ENSO index (-1.0) (MinCent) and incorporating the observed ENSO OI indices 2 and 4 months prior to and post the observed minimum.

We used BNNs to predict crop cacao yield when trained on data from the on-farm trials conducted across Sulawesi.

## Results

### Climate data

**Figure 1:**
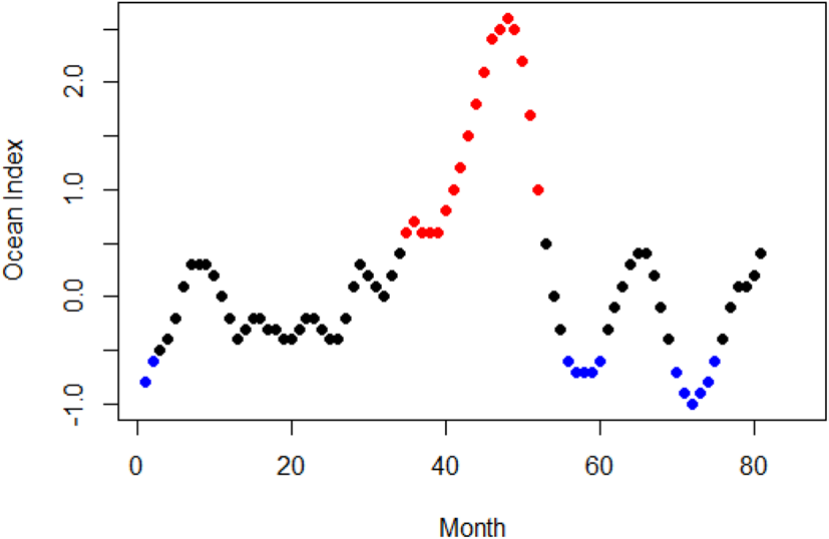
Three month running means of ENSO Oceanic indices (ENSO OI) from February 2012 until October 2018. Points with a strong negative score (< − 0.5) are colored blue while points with a strong positive score (> 0.5) are colored red.

The ENSO OI 3-month running means ranged from − 1 up to 2.6 (Fig. 1).

### Bayesian neural network construction

A key step in the development of a neural networks is to decide on the architectural structure of the network. For the study reported here, the number of outputs was fixed as a single node representing the dependent variable (crop yield), while the number of input nodes was fixed at the number of independent variables available for analysis. An initial experiment was conducted to identify an appropriate architecture for the hidden layer and to examine the anticipated precision of the constructed networks using tenfold cross validation (Table 1). The yields predicted from optimally constructed BNNs were highly accurate when conditioned using both long and short-term data, with models derived from both ENSO profiles accounting for up to ¾ of all variation within the cacao yield (Table 1).

**Table 1:** Architecture of the hidden layer and network precision for Bayesian neural networks constructed to predict cacao crop yield using either short term (1–9 months) or long term (1–25 months) ENSO data.

ENSO profile	Nodes within the hidden layer nodes	Mean square error	r^2^
Short term ENSO	9	270.8	0.692
Long term ENSO	12	143.5	0.768

Given that both models returned similarly high levels of precision, we utilised the simpler network based on the short-term ENSO profile for subsequent analysis.

Detailed analysis of the error observed during tenfold cross validation of the BNN based on the short term ENSO profile shows a strong linear relationship between observed cacao yields and those predicted by the BNN (Supplementary Fig. S.1) and the ENSO OI during crop development had no discernible impact on the network precision. Linear regression showed this relationship to be highly significant (Supplementary Table S.2), with an intercept close to zero but a constant slightly less than 1. An examination of the errors observed within each group during cross-fold validation shows that the mean and median absolute prediction errors within group were very close to zero, and that the majority of errors were less than + / − 10 kg ha^−1^ month^−1^, indicating that all groups returned a similarly high level of precision.

### Cacao crop yield prediction

We utilized the models to predict the impact of contrasting ENSO OI profiles on crop yield and response to fertilizer applications. The difference between the accuracy of the simpler short and more complex long-term ENSO profiles was small with a slight gain with the more complex profile.

**Figure 2:**
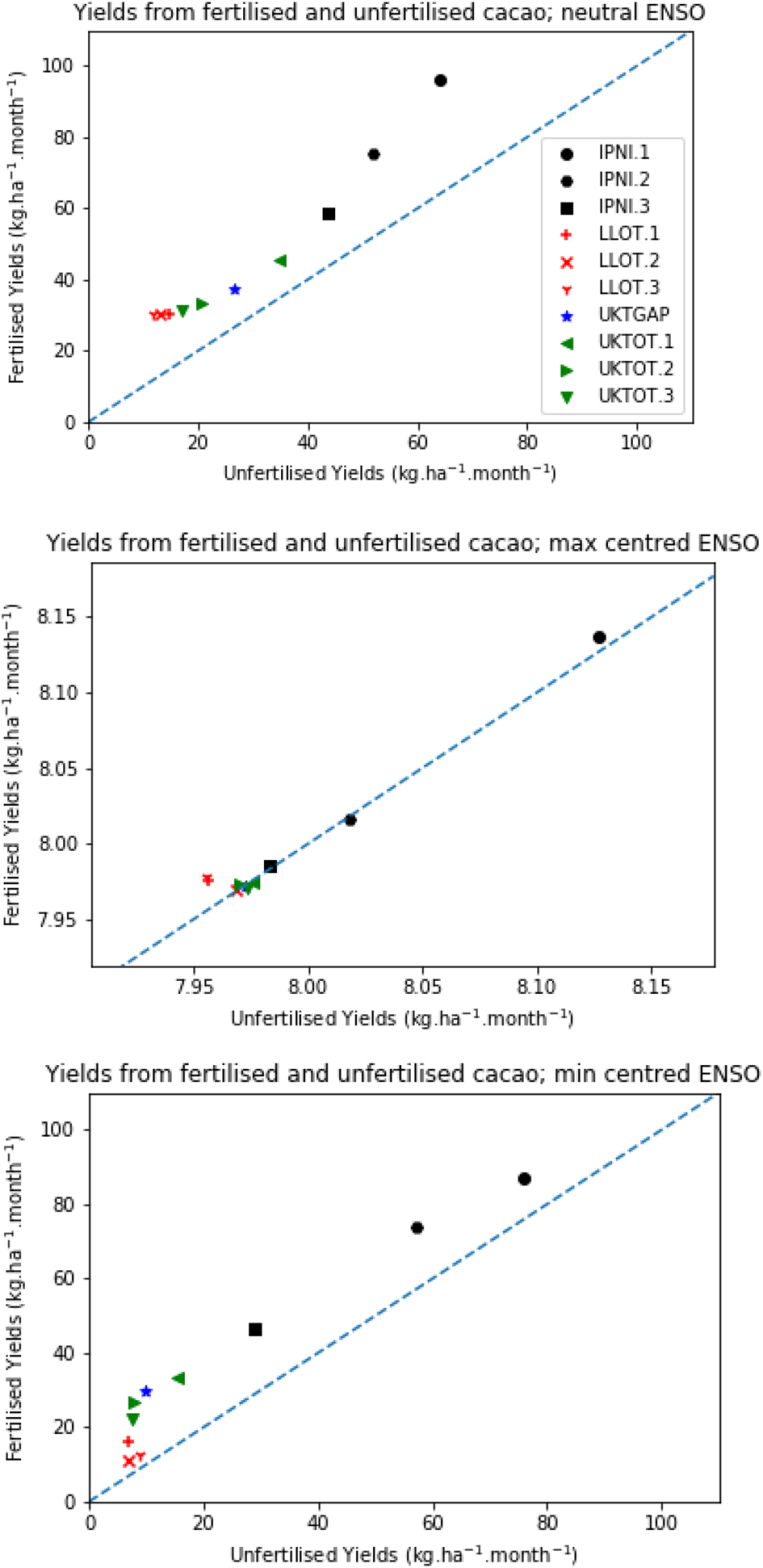
Most likely predicted cacao yields from fertilized and unfertilized crops under three contrasting ENSO OI profiles over the 9 months prior to harvest: (**a**) continuous neutral ENSO OI conditions; (**b**) ENSO OI conditions centred around the maximum observed value; and (**c**) ENSO OI conditions centred araound the minimum observed value. The dashed blue line indicates a 1:1 relationship between fertilized and unfertilized yields. Note the differente scale utilised for plot (**b**) (ENSO OI conditions centred around the maximum observed value).

The hypothetical ENSO profiles have a major impact on predicted cacao production (Fig. 2, Fig. S.1). Under the *Neutral* profile, predicted yield for unfertilized cacao crops was between 12 and 64 kg ha^−1^ per month, with large differences in the productivity of the distinct farmers` groups. Yields under the *MinCent* profile were somewhat similar, ranging from 7 up to 76 kg ha^−1^ per month with similar group rankings. Predicted productivity was extremely low for all groups under the *MaxCent* profile: all groups yielded less than 8.2 kg ha^−1^ per month. The range of responses to weather events was linked to group productivity; IPNI.1 gave the greatest predicted unfertilized yield under *Neutral* conditions and also displayed the greatest predicted yield changes under different ENSO OI profiles (67.6 kg ha^−1^ per month). In contrast, LLOT.3 had the lowest predicted unfertilized yield under neutral conditions and productivity was reduced by only 3.8 kg ha^−1^ per month under the *MaxCent* profile.

The cacao crop showed a strong fertilizer response for all groups under the *Neutral* ENSO OI profile, and the response tended to be greater within those groups that showed the highest productivity under unfertilized conditions (Fig. 2). Most groups gave a strong yield response to fertilizer under the *MinCent *ENSO OI profile, but the response was particularly reduced for three low yielding groups (LLOT.1, LLOT.2 and LLOT.3). All yields were particularly low under the *MaxCent *profile, with no discernible response to fertilizer applications.

The standard deviation among predictions was very low across all groups and ENSO profiles, ranging from a minimum of 0.394 kg ha^−1^ month^−1^ (IPNI.1 under the maximum centered ENSO profile) up to 0.696 kg ha^−1^ month^−1^ (IPNI.3 under the continuous neutral ENSO profile) (Table S.3).

## Discussion

The BNNs demonstrated that the average yields of cacao farmer groups, in Sulawesi over distinct time periods, are closely associated with the ENSO OI patterns 9 to 25 months before harvest. The ENSO OI short term pattern explained slightly less (69%) of the variation in the average yield than the long term pattern (77%). We consider both these levels of prediction to be high, however, the short term pattern level was simpler and was used for further analyis. The linear regression between predicted and actual yields indicates that the model will tend to underestimate cacao productivity at high yields (e.g. in excess of 100 kg ha^−1^ month^−1^).

The predictions made by the BNNs indicated that cacao yields are substantially impacted by ENSO conditions, which accords with prior observations^21^. The fertilizer response varied according to the ENSO profile: the greatest predicted response was in the *Neutral* ENSO profile with a smaller response under the *MinCent* ENSO profile, especially when unfertilized yields were low, and essentially no response under the *MaxCent* ENSO profile. Hence, the analysis provides insights into the appropriate fertilizer regime for distinct ENSO OI patterns in the period 9 months before harvest. We also note that recent methods to improve prediction of future ENSO OI patterns make it possible to predict them with reasonable accuracy for up to 1 year^3^. Thus, it is possible to relate average cacao crop performance and management practices directly to ENSO patterns in a given region without the need for weather data when the following conditions are met: (1) data exist on crop performance in any given site over time with distinct management practices; and (2) the weather patterns are driven by ENSO OI. We have used cacao as proof-of-principle, and suggest that this principle can readily be applied to other crops.

A great advantage that Bayesian methods have over other machine learning approaches is that they can utilise variance based probability distributions to predict the likelihood of any given outcome. The model was used to predict the most likely monthly yield and expected standard deviation from each farm group under a specific ENSO profile when either fertilized or unfertilized. The standard deviations attained across all predicted responses was remarkably low, typically less than 1 kg ha^−1^ per month. Both the construction of the model and the subsequent predictions were based upon the mean yield data from 10 farms in each group at each monthly harvest under a single management type. As a result, all variations in yield across those 10 farms would have been excluded from the network constructed. As a consequence, while the predictions returned by the model might precisely reflect the mean response from each group, the limited input data will mean that the range of possible outcomes under any predicted scenario is likely to be underestimated. Up to now we have established proof-of-principle stage, the next stage will be first to improve the assessment of the predicted probability distributions and then to develop channels for communicating the results of the analysis to farmers followed by appraisal of their opinions and use of the information provided. Options for improving estimates of the probability distribution include both incorporating all observations from within each group, to ensure that farm-to-farm variance is adequately captured, and to extend the observations across more seasons to ensure that the variability of response to contrasting ENSO profiles is better represented.

The analysis presented here is based on the average yields for each group of farmers. However, previous analysis indicates much variation in yield within the farmers groups^20^. Furthermore, those farmers with higher average yields tended to maintain their yield advantage relative to those with lower yields, even when conditions were adverse. This supports the view that the differences in yield between the high average yield and the low average yield farmers are due to management skills, rather than more favorable soils and weather conditions^20^. This suggests that if the average yields of individual farmers relative to the mean of all farmers are known, then the ENSO predictions can be used to predict their yield levels, and also their response to fertilizer applications.

The demonstration that on farm yields and response to one management variable, fertilizer, can be linked directly to ENSO OI data supports the view that, in the future, with cacao or other crops, data on farm yields obtained with distinct management practices can be coupled with ENSO OI data to both determine probable crop yields and also to define differential crop response to management at specific sites under distinct ENSO OI patterns without the need for accurate weather data. The ENSO OI data exists, what is often lacking is data on yield with distinct management practices. To obtain this type of information in heterogeneous growing environments using traditional Randomized Control Trials is simply not possible. However, we suggest that schemes, such as those to collect the cacao data we have here with distinct management treatments superimposed on farmers fields^20^, can be used. Furthermore, even without superimposing management practices, simply monitoring crop performance, weather and the variation in management practices of farmers can be used to relate yield to variation in weather patterns and management^28–30^. However, this is only effective if the data of a large number of cropping events is brought together for analysis, which requires social organization and the willingness to share data^28^. Our experience with cacao indicates that small farmers are willing to share data, but an external agency is required to manage the overall process of data collection and compilation^20^. Similar experiences with CropCheck and in Australia and Chile support this point of view^31,32^. The value of shared information through formation of farmer groups is well established^33,34^ and we suggest that the methodology described here could be implemented through farmer groups. Hence, through monitoring of crop performance and management coupled with Bayesian based machine learning tools and currently available ENSO OI information and predictions, farmers and agronomists can adjust management practices, in this case fertilizer applications, according to ENSO profiles. This will require social organization and support for the collection, compilation and analysis of the data; however, we believe it offers a route to provide farmers with an improved and cost effective knowledge base, derived from sparse data resources, to better manage their crops.

Social organization is not only required for the collection of data to be analysed, but also for the disemination to farmers of the knowledge generated though its interpretation. Current tendencies of providing farmers with the basis to make better decisions recognise the restrictions of the linear model for extension and tend towards active farmer participation in the interpretation of data through such mechanisms as farmers field schools^35^, formation of farmers groups (see for example Montaner 2004^34^) and innnovation networks (see for example Klerkx et al. 2010^36^, Wood et al. 2014^37^, World Bank, 2008^38^). Further development of farmers´organizations and innovation networks will be required to effectively deploy the concepts presented in this paper.

The principles developed here could be applied to other crops, such as coffee, olive and oil palm, and this type of analysis could be extended to other regions, such as Africa where data on crop response to management and weather variation is sparse. At the same time, we note that additional information on, *inter alia*, crop management, topography and soil types could substantially improve the predictive power of the networks. Furthermore, these machine learning techniques can be used to mine existing big data sets collected by large commercial interests, to discover relationships between environment, management and crop production, and thereby supplement, at low cost, the findings generated by formal controlled scientific experiments. In the case of small farmers, social organization and external support will be required.

There are several caveats on the use of this proposed methodology. First, the relationship between the ENSO phenomenon and the weather patterns will be specific to each location or recommendation domain. Hence, models and inferences for management cannot be readily transferred from one recommendation domain to another. Furthermore, the definition of the area that comprises a recommendation domain is not simple. Thus, whilst we consider the principles developed here to be universal, the models themselves will be specific to each recommendation domain, which are currently still difficult to define but new approaches are becoming increasingly available to do so (e.g. Rubiano et. al. 2016^18^; Rattalino Edreira et al. 2018^17^).

A further complication of the suggested approach is the lack of understanding of the underlying mechanisms that establish the associations. This deficiency limits the ability to identify the specific causes of different crop productivities, and thus limits our ability to resolve these unidentified problems.

Growers decisions on how much to invest in their crop production practices depends on the expected prices of the commodities they produce: when prices are expected to be high, they will invest more, and when prices are low they may even abandon their crops. It has not escaped our notice that the predictive power of the machine learning resources would also provide the cacao industry as a whole with insights into the fluctuations in future cacao supply and hence prices. This would allow farmers and others in the cacao supply chain to minimize uncertainty and better manage the overall industry. The experiences strongly support the idea that machine learning is a useful tool in our armoury opening the opportunity to utilize information from on farm performance coupled with publicly available data to improve agricultural management.

## Conclusions

We show that if time series data is available on both yield and management practices, even in the absence of weather data for the location, yield and response to management practices can be predicted from the state of the ENSO phenomenon. We note that the prediction models are not explicative and are only valid for one crop and one specific recommendation domain in areas where the ENSO is a major driver of weather variability. Nevertheless, monitoring of crop performance on farms with distinct management practices over time, when linked to the ENSO data, can throw light on how to better manage crops. This methodology is particularly apt for crops for which conventional research approaches using controlled trials are limited, when simulation models do not exist and in areas where weather data is sparse.

## Supplementary Information

Supplementary Information.
