# Supplementary material for: Crop response to El Niño-Southern Oscillation related weather variation to help farmers manage their crops
Source: Sci Rep. 2021 Apr 15;11:8292. doi: 10.1038/s41598-021-87520-4 (PMC8050235; doi:10.1038/s41598-021-87520-4)
Supplement: Supplementary file 1 — Supplementary Information. [file 41598_2021_87520_MOESM1_ESM.docx]

Crop response to El Niño-Southern Oscillation related weather variation to help farmers manage their crops

Supplementary Information

Ross Chapman^1*^, James Cock^2^, Marianne Samson^3^, Noel Janetski^4^, Kate Janetski^5^, Dadang Gusyana^6^, Sudarshan Dutta^7^ and Thomas Oberthür^8^

^1^Data Analysis Consultant, 9 Kia Ora Parade, Ferntree Gully, Victoria 3156, Australia. Email: [rosspjchapman@gmail.com](mailto:rosspjchapman@gmail.com)

^2^Emeritus, Centro Internacional de Agricultura Tropical (CIAT), Cali, Columbia. Email: [jamescock@gmail.com](mailto:jamescock@gmail.com)

^3^ Scientific Data Management Consultant, Los Baños, Philippines, [marianne@samson.ph](mailto:marianne@samson.ph)

^4^ Senior Technical Advisor to the Cocoa Care Program of Community Solutions International, Makassar, Sulawesi, Indonesia, [noel.janetski@gmail.com](mailto:noel.janetski@gmail.com)

^5^ CEO of Community Solutions International, Bali, Indonesia, [kjanetski@gmail.com](mailto:kjanetski@gmail.com)

^6^ Agronomy Manager, Lautan Luas TBK, Jakarta, Indonesia, [dadang.gusyana@lautan-luas.com](mailto:dadang.gusyana@lautan-luas.com)

^7^Scientist, African Plant Nutrition Institute, Lot 660, Hay Moulay Rachid, Ben Guerir, Morocco. Email: [S.Dutta@apni.net](mailto:S.Dutta@apni.net)

^8^Director, Business & Partnership Development, African Plant Nutrition Institute, Lot 660, Hay Moulay Rachid, Ben Guerir, Morocco. Email: [t.oberthur@apni.net](mailto:t.oberthur@apni.net)

* - Corresponding author.

Table S.1 Monthly mean monthly El Nino Southern Oscillation Ocean Index (ENSO OI) values during the months leading up to harvest for three contrasting ENSO OI profiles utilised to predict the impact of fertilizer management on cacao yields under contrasting weather conditions; continuous neutral ENSO OI conditions (*Neutral*); an ENSO profile based on observed ENSO values in a period that spanned the maximum observed ENSO index (2.6) (MaxCent); An ENSO profile based on observed ENSO values in a period that spanned the minimum observed ENSO index (-1.0) (MinCent)

| Month prior to harvest ENSO OI from which the recorded Months prior | | 9 | 7 | 5 | 3 | 1 |
| --- | --- | --- | --- | --- | --- | --- |
| ENSO OI Profile name | Neutral | 0 | 0 | 0 | 0 | 0 |
|  | MaxCent | 1.8 | 2.4 | 2.6 | 2.2 | 1.0 |
|  | MinCent | -0.1 | -0.07 | -1 | -0.8 | -0.4 |

Figure S.1

a


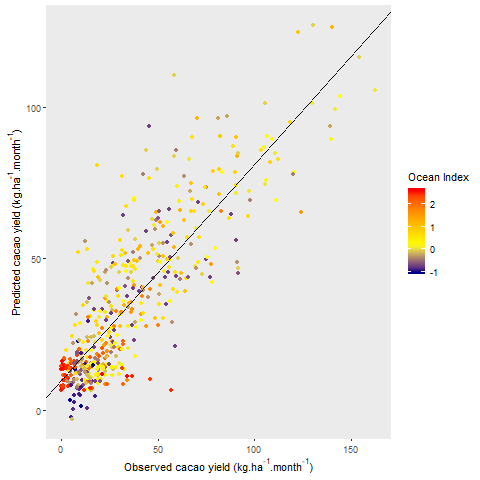


b


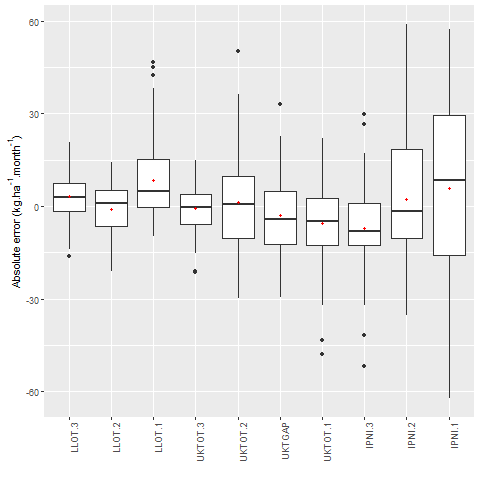


Figure S.1 A summary of absolute errors recorded for every data point used in the testing of the Bayesian Neural Network using 10-fold cross validation: a) a scatterplot of predicted cacao yields against actual cacao yields with data points coloured by El-Nino Southern Oscillation Ocean Index 4 months prior to harvest; b) the range of absolute errors recorded for each data point within each farmer group – median error values are represented by the central horizontal bar while the mean error values are represented by the red dot. Individual dots represent outliers beyond the percentiles.

Table S.2. A summary of the linear regression of coefficients and statistics for linear modelling of all the predicted against observed cacao yields using all data points predicted during the 10-fold cross validation of the Bayesian Neural network derived from the short term EL-Nino Southern Oscillation Index Profile.

| Intercept | Coefficient | r^2^ | p-value |
| --- | --- | --- | --- |
| 10.1 | 0.71 | 0.7 | p<0.001 |

Table S.3 Standard deviation values for the cacao yield predicted (kg ha^-1^) under three contrasting ENSO OI profiles over the 8 months prior to harvest for 10 groups homologous farms.

| Farmer group | Standard deviations for predictions made under continuous neutral conditions | Standard deviations for predictions made under ENSO OI conditions centred around the maximum observed value | Standard deviations for predictions made under ENSO OI conditions centred around the minimum observed value |
| --- | --- | --- | --- |
| IPNI.1 | 0.658 | 0.394 | 0.486 |
| IPNI.2 | 0.670 | 0.400 | 0.572 |
| IPNI.3 | 0.696 | 0.396 | 0.563 |
| LLOT.1 | 0.395 | 0.398 | 0.552 |
| LLOT.2 | 0.399 | 0.400 | 0.568 |
| LLOT.3 | 0.393 | 0.398 | 0.656 |
| UKTGAP | 0.433 | 0.397 | 0.469 |
| UKTOT.1 | 0.550 | 0.399 | 0.476 |
| UKTOT.2 | 0.409 | 0.400 | 0.479 |
| UKTOT.3 | 0.399 | 0.394 | 0.520 |
